# Sex-Specific Gene-by-Vitamin D Interactions Regulate Susceptibility to Central Nervous System Autoimmunity

**DOI:** 10.3389/fimmu.2018.01622

**Published:** 2018-07-17

**Authors:** Dimitry N. Krementsov, Loredana Asarian, Qian Fang, Mahalia M. McGill, Cory Teuscher

**Affiliations:** ^1^Department of Biomedical and Health Sciences, University of Vermont, Burlington, VT, United States; ^2^Department of Medicine, University of Vermont, Burlington, VT, United States; ^3^Department of Pathology, University of Vermont, Burlington, VT, United States

**Keywords:** vitamin D, multiple sclerosis, CD4 T cells, genetic variation, experimental autoimmune encephalomyelitis (EAE), sex differences, gene–environment interactions, wild-derived inbred strains

## Abstract

Vitamin D3 (VitD) insufficiency is postulated to represent a major modifiable risk factor for multiple sclerosis (MS). While low VitD levels strongly correlate with higher MS risk in white populations, this is not the case for other ethnic groups, suggesting the existence of a genetic component. Moreover, VitD supplementation studies in MS so far have not shown a consistent benefit. We sought to determine whether direct manipulation of VitD levels modulates central nervous system autoimmune disease in a sex-by-genotype-dependent manner. To this end, we used a dietary model of VitD modulation, together with the autoimmune animal model of MS, experimental autoimmune encephalomyelitis (EAE). To assess the impact of genotype-by-VitD interactions on EAE susceptibility, we utilized a chromosome substitution (consomic) mouse model that incorporates the genetic diversity of wild-derived PWD/PhJ mice. High VitD was protective in EAE in female, but not male C57BL/6J (B6) mice, and had no effect in EAE-resistant PWD/PhJ (PWD) mice. EAE protection was accompanied by sex- and genotype-specific suppression of proinflammatory transcriptional programs in CD4 T effector cells, but not CD4 regulatory T cells. Decreased expression of proinflammatory genes was observed with high VitD in female CD4 T effector cells, specifically implicating a key role of MHC class II genes, interferon gamma, and Th1 cell-mediated neuroinflammation. In consomic strains, effects of VitD on EAE were also sex- and genotype dependent, whereby high VitD: (1) was protective, (2) had no effect, and (3) unexpectedly had disease-exacerbating effects. Systemic levels of 25(OH)D differed across consomic strains, with higher levels associated with EAE protection only in females. Analysis of expression of key known VitD metabolism genes between B6 and PWD mice revealed that their expression is genetically determined and sex specific and implicated *Cyp27b1* and *Vdr* as candidate genes responsible for differential EAE responses to VitD modulation. Taken together, our results support the observation that the association between VitD status and MS susceptibility is genotype dependent and suggest that the outcome of VitD status in MS is determined by gene-by-sex interactions.

## Introduction

Multiple sclerosis (MS) is a multifactorial autoimmune disease, in which an immune-initiated attack on the central nervous system (CNS) results in demyelination, axonal loss, and eventual neurological dysfunction. Genetics contribute to a significant portion of MS risk, with estimates ranging from 20 to 30% (1). The primary genetic risk factor lies in the major histocompatibility complex class II locus, with up 200 other minor risk loci identified by recent genome-wide association studies (2).

The remainder of MS risk is thought to come from environmental factors or gene-by-environment interactions. A number of different environmental risk factors have been associated with MS susceptibility. The most prominent of these are Epstein–Barr virus (EBV) infection, low sunlight/ultraviolet (UV) radiation exposure, vitamin D3 (VitD) deficiency, and cigarette smoking (3, 4). In addition, over the past 100 years, MS incidence has remained stable in men, but has tripled in women, suggesting the existence of sex/gender-specific risk factors and/or behavioral changes (3).

Vitamin D3 is one of the best-studied MS risk factors. Early epidemiologic studies documented a gradient of increasing MS incidence with increasing distance from the equator, which in later studies was attributed to decreased exposure to sunlight/UV radiation (3). This protective effect of sunlight in MS has long been thought to be mediated by the immunomodulatory effects of VitD. Photoconversion of 7-dehydrocholesterol to VitD (cholecalciferol) in the skin is catalyzed by UV-B radiation. VitD from the skin and dietary VitD (absorbed in the intestine) enters the circulation and is subsequently converted in the liver to calcidiol [25(OH)D_3_], and then in the kidney or in target tissues to calcitriol [1,25(OH)_2_D_3_], the hormonally active form which can bind and activate the nuclear vitamin D receptor (VDR) in many different target tissues, including bone, kidneys, intestine, and the immune system (5). Calcitriol-mediated activation of VDR in different immune cells is thought to generally result in immunoregulatory transcriptional responses (5). With regard to MS, low systemic VitD levels [typically measured using the most stable VitD metabolite 25(OH)D_3_ as a surrogate] are associated with increased disease risk (6), relapse rate, and disease progression (7, 8). A number of MS susceptibility genes are predicted to be regulated by VitD (9, 10), but the underlying *in vivo* mechanisms contributing to the etiopathogenesis of MS remain unclear. In addition, recent Mendelian randomization studies have shown that genetic variants that are associated with reduced circulating 25(OH)D_3_ levels also predict increased risk of MS (11–13). There are ongoing clinical trials to test the benefits of dietary VitD supplementation as a preventative or therapeutic strategy, but to date no clear beneficial effect has been reported (14–17). Importantly, the immunosuppressive effects of UV radiation independent of VitD are also well documented (18, 19). In fact, the results of recent epidemiological studies suggest that VitD and UV radiation can exert independent effects on MS risk (20–22), and data from animal models support this concept (19, 23–25).

Intriguingly, while low VitD levels are strongly associated with MS risk in white populations, a number of studies have shown that this is not the case in blacks and Hispanics (6, 26–30). This is surprising, since these populations typically have darker skin pigmentation and thus lower VitD levels compared with whites living at the same latitude (31), and it demonstrates that the association between VitD and MS risk is modified by unidentified genetic factors. This also suggests that any benefits of VitD supplementation for MS would be genotype dependent.

Effects of VitD have also been explored in animal models of MS. Experimental autoimmune encephalomyelitis (EAE), the principal autoimmune model used to study the pathogenesis of MS, can be induced by immunization with CNS homogenate or specific myelin proteins/peptides, or by transfer of CD4 T cells reactive to these antigens (32). As in MS, autoreactive CD4 T cells enter the CNS to initiate inflammation and pathology, culminating in neurologic disability. Treatment of adult animals with the hormone calcitriol has long been known to suppress EAE in mice (33, 34). More recent mechanistic studies have shown that this suppression requires VDR signaling in T cells and the expression of interferon gamma (IFNγ) (35, 36), thus likely acting directly to inhibit T helper (Th)-1 effector functions. In addition, calcitriol-mediated suppression of EAE is associated with induction of regulatory T cells (Tregs) (37), and hence it has been proposed that VDR signaling may act as a switch between Th1 effector and regulatory CD4 T cells (38). However, the physiologic validity of this approach has been called into question, as treatment with calcitriol can cause hypercalcemia, which itself can suppress EAE (39, 40), although this point remains controversial (38). As a more physiologically relevant approach, dietary supplementation with VitD during adolescence also inhibited EAE in mice (41, 42) and rats (43, 44). Strikingly, this effect was observed only in female, but not male mice, and was dependent on the presence of estrogen (41, 42).

The EAE model is an attractive approach to directly test hypotheses generated from epidemiologic studies on MS risk factors (45). However, one of the common limitations of this approach is that the immense genetic diversity of human populations is not represented among standard inbred laboratory strains of mice (46, 47). We and others have attempted to more closely model human genetic diversity by incorporating into the experimental design wild-derived inbred strains of mice, such as PWD/PhJ (PWD), or chromosome substitution (consomic) strains that carry individual PWD chromosomes on the common B6 background (B6.Chr^PWD^) (48, 49). We have shown that compared with B6 mice, a classical inbred laboratory strain, wild-derived inbred PWD mice have widely divergent immune cell transcriptomes, which result in differential expression of MS relevant genes and reduced susceptibility to EAE (50). We have also used the B6.Chr^PWD^ consomic model to identify multiple EAE susceptibility loci, many of which were sex specific (51).

In this study, we combined the physiologically relevant dietary approach to manipulate VitD levels (41–43), with the genetic diversity of B6.Chr^PWD^ consomic model, to examine gene-by-sex interactions on the effects of VitD in CNS autoimmune disease. In agreement with previous reports, we show that the effects of VitD supplementation on EAE susceptibility in B6 mice are female specific. This was associated with induction of sex- and genotype-specific transcriptional responses in effector and regulatory CD4 T cells. Strikingly, the EAE response to VitD supplementation varied widely across B6.Chr^PWD^ consomic strains, suggesting that, as in MS, genotype modifies the outcome of VitD status in EAE.

## Results

### Dietary Manipulation of VitD Levels Modulates Systemic VitD Levels and EAE Severity

**Figure 1:**
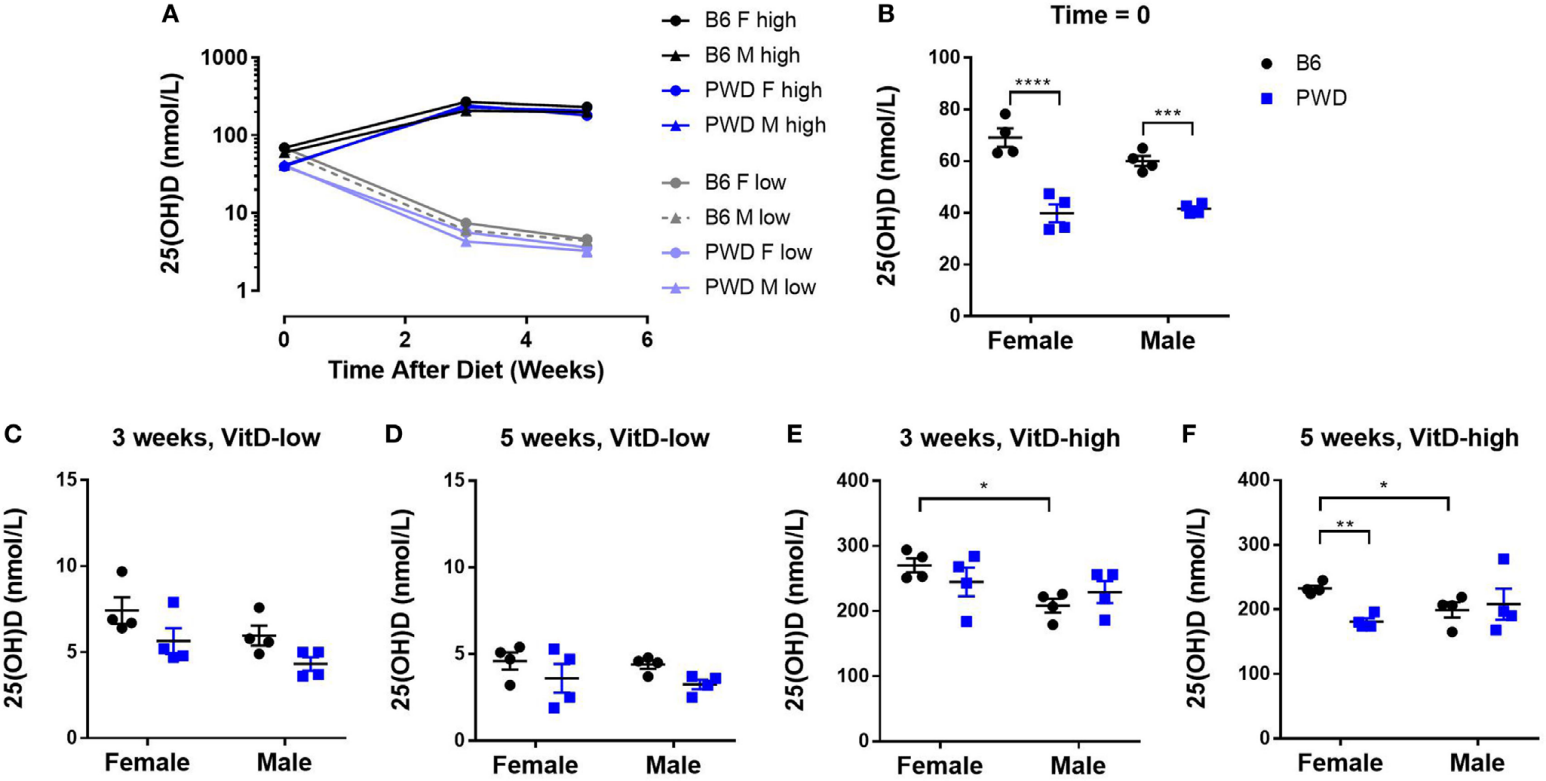
Manipulation of dietary vitamin D3 (VitD) results in robust changes in systemic VitD levels that are sex- and genotype dependent. Female and male B6 and PWD mice (*N* = 4 for each sex/strain combination) were assigned to VitD-high and VitD-low diets at 3 weeks of age. Serum samples were collected at the outset (Time = 0), and at 3 and 5 weeks post-treatment. 25(OH)D levels were measured by enzyme-linked immunoassay (see Materials and Methods). An overview of kinetic data is shown in **(A)**, followed by comparisons at individual time points in **(B–F)**, segregated by diet. The significance of the observed differences in **(B–F)** was assessed by two-way ANOVA, with Holm–Sidak’s *post hoc* comparisons: B6 vs. PWD (within sex), and female vs. male (within strain). Symbols indicate a significant difference between the indicated groups, as follows: *p < 0.05; **p < 0.01; ***p < 0.001; ****p < 0.0001.

To directly manipulate systemic VitD levels in a controlled fashion, we adopted a previously described dietary paradigm initiated during adolescence, which has been shown to modulate EAE in the mouse (41, 42) and rat (43, 44). Briefly, 3-week-old B6 and PWD mice were randomized to either a VitD-low diet or a VitD-high diet, as described in Section “Materials and Methods” (see Figure S1 in Supplementary Material for a diagrammatic overview of the experimental design). Serum samples were collected at 3 and 5 weeks post-dietary intervention, and analyzed for the levels of 25(OH)D, the most abundant and stable metabolite of VitD that is typically used as an indicator of VitD status in clinical studies (6). The dietary regimen induced rapid and sustained changes that appeared to plateau at approximately 3 weeks post-intervention (Figure 1A). PWD mice exhibited significantly lower baseline 25(OH)D levels compared with B6 (Figure 1B). These differences disappeared on the VitD-low diet (Figures 1C,D). Interestingly, on the VitD-high diet, female B6 mice reached higher levels of 25(OH)D compared with B6 males at both time points (Figures 1E,F), which were also significantly higher than PWD females at 5 weeks (Figure 1F).

**Figure 2:**
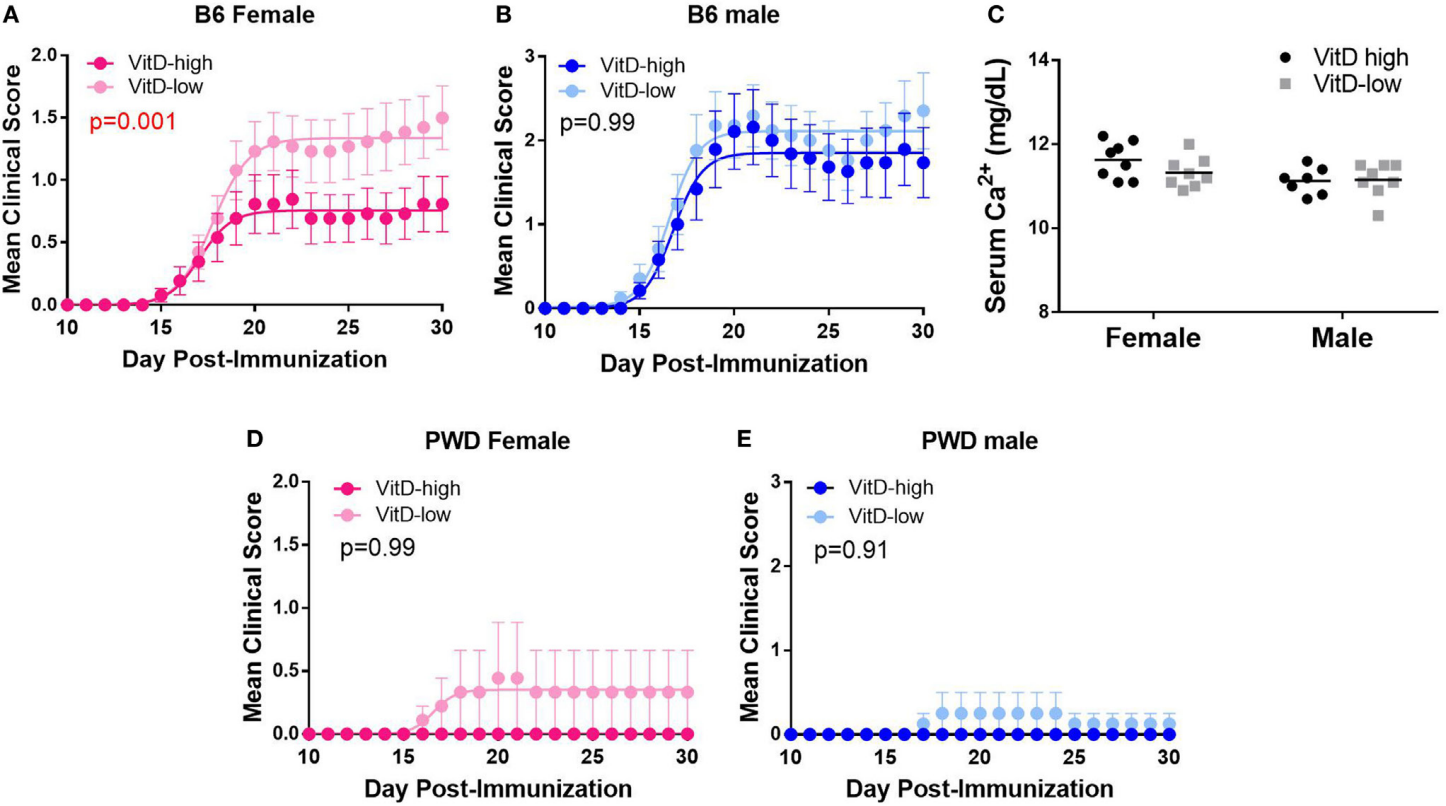
Strain- and sex-specific suppression of EAE by vitamin D3 (VitD). B6 **(A–C)** and PWD **(D,E)** mice were maintained on VitD-high and VitD-low diets as in Figure 1, followed by EAE induction after 5 weeks post-dietary intervention. Mice were maintained on the respective diets until the end of the experiment. **(C)** B6 serum Ca^2+^ levels were measured for the indicated groups, and the significance of the observed differences assessed by two-way ANOVA, followed by Holm–Sidak’s *post hoc* comparisons. The significance of the observed differences in the severity of the clinical disease course was assessed as described in Section “Materials and Methods.” *p*-Values for the effect of diet on overall disease course (representing the interaction term for treatment × time, repeated measures two-way ANOVA) are shown. The numbers of animals for each group are provided in Table S1 in Supplementary Material.

Having established the dietary paradigm, we tested whether the robust differences in systemic VitD levels achieved by VitD-high and VitD-low diets impacted clinical progression of EAE. Female and male B6 mice were subjected to the dietary paradigm above, followed by EAE induction at 5 weeks post-dietary intervention. Exposure to the VitD-high diet resulted in lower EAE severity compared with VitD-low diet in female B6 mice (Figure 2A). By contrast, no significant difference in EAE clinical course on the two diets was observed in male B6 mice (Figure 2B). DeLuca and colleagues have observed that suppression of EAE in mice by treatment with the bioactive VitD metabolite, calcitriol, is associated with and dependent on hypercalcemia, questioning the physiological relevance of that approach (39, 40). Hence, we tested whether our dietary paradigm affected systemic Ca^2+^ levels and found that EAE suppression was not accompanied by significant changes in Ca^2+^ levels (Figure 2C), suggesting that our dietary model influences EAE susceptibility independent of Ca^2+^.

We have previously shown that PWD mice are almost completely resistant to EAE (50). To test whether this resistance could be affected by VitD status, PWD mice were subjected to the dietary regimen as above, followed by EAE induction. VitD had no significant effect on EAE, whereby PWD mice remained highly resistant to EAE induction on either diet (Figure 2D; EAE incidence: VitD-high 0/8, VitD-low 2/15; *p* = 0.53). Taken together, our results demonstrate that high VitD provides female-specific protection from EAE in susceptible B6 mice, in agreement with previous reports (41, 42).

### *In Vivo* Modulation of CD4 T Cell Transcriptome by VitD Is Genotype-, Sex-, and Cell Type Specific

**Figure 3:**
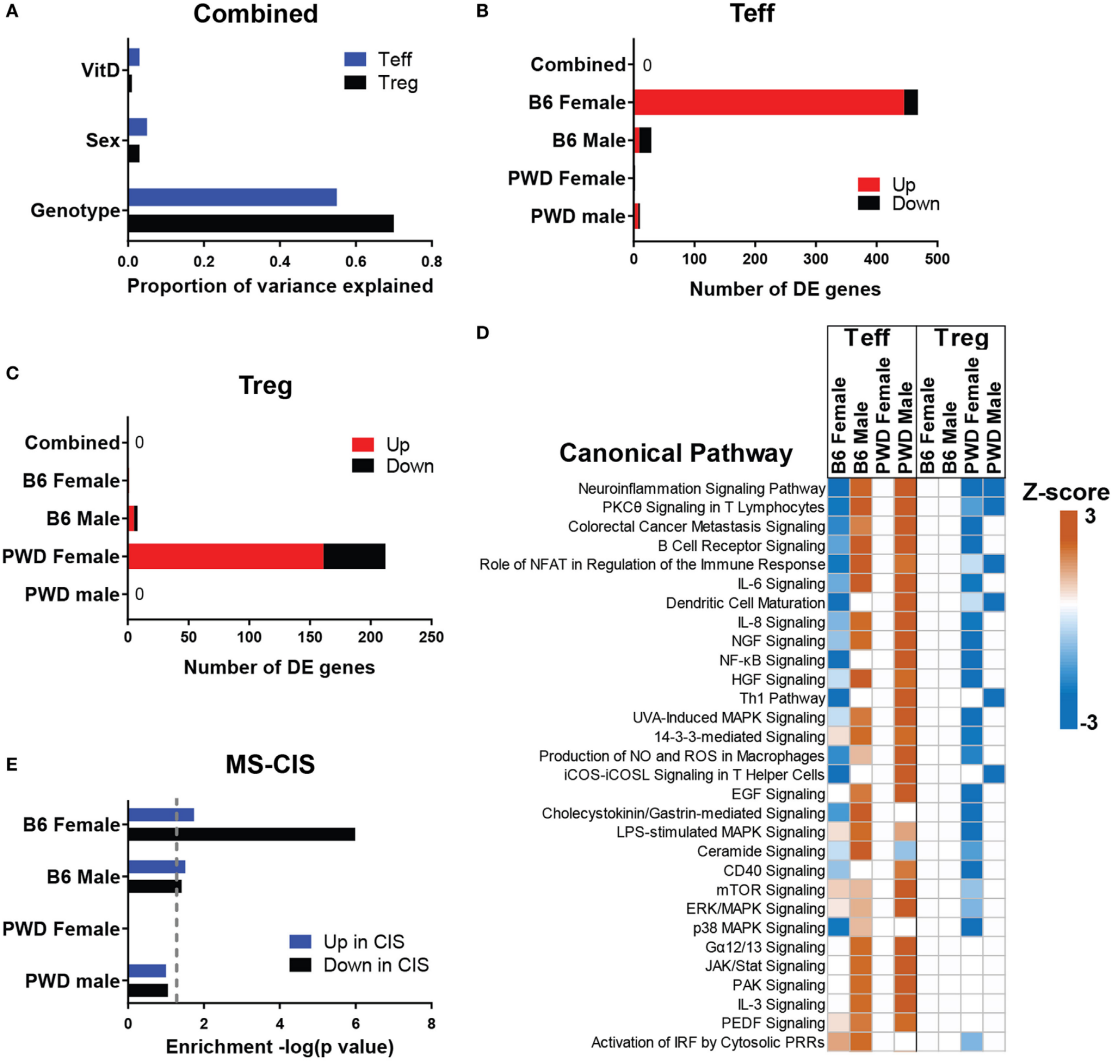
*In vivo* transcriptional regulation by vitamin D3 (VitD). Female and male B6 and PWD mice (*N* = 4 for each sex/strain combination) were exposed to VitD-low and VitD-high diets as in Figure 1. At 5 weeks post-dietary intervention, transcriptional profiling was performed on Teff and regulatory T cells (Tregs) as described in Section “Materials and Methods.” **(A)** The proportion of variance in gene expression explained by the indicated experimental variables is shown for Teff and Tregs. **(B,C)** The number of genes passing the differential expression threshold (|Fold Change| > 2, ANOVA *p* < 0.001) as a function of diet is shown for the indicated groups, in Teff **(B)** and Tregs **(C)**. Direction of change is depicted as expression level in VitD-high relative to VitD-low (e.g., “up” indicates higher expression in the VitD-high group). **(D)** Canonical pathway analysis was performed using IPA software (see Materials and Methods). *Z*-scores indicate predicted direction and relative strength of change (VitD-high relative to VitD-low). The top 30 pathways (ranked by *Z*-score) are shown. **(E)** Enrichment analysis of the indicated differentially expressed (DE) gene sets from Teff within transcripts DE (up or down) in CD4 cells from multiple sclerosis (MS)-CIS patients was performed using IPA (see Materials and Methods). *p*-Values for enrichment are shown as −log(*p*). Dotted line denotes *p* = 0.05.

CD4 T cells are thought to initiate the inflammatory cascade in MS. Studies in EAE suggest that these cells are also the most likely target of VitD, either by modification of function of CD4 effector T cells (Teff) or by induction of regulatory CD4 T cells (Tregs) (38). Hence, to understand the molecular mechanisms underlying immune modulation by VitD *in vivo*, we carried out transcriptional profiling of Teff and Tregs isolated from B6 and PWD mice exposed to VitD-low or VitD-high diets. Mice were subjected to the dietary paradigm as described in Figure 1, followed by isolation of splenic Teff and Tregs using fluorescence-activated cell sorting (FACS) and transcriptional profiling (see Materials and Methods) at 5 weeks post-dietary intervention (Figure S1 in Supplementary Material). When gene expression data were analyzed for effect of genotype, sex, and VitD, it was found that genotype accounted for the largest effect size, with modest effects of sex and VitD (Figure 3A). This is consistent with our published data showing strikingly divergent transcriptomes between B6 and PWD immune cells (50). Consistent with these observations, when both strains and sexes were pooled and analyzed together for effect of VitD, very few differentially expressed (DE) genes were detected in either cell type (Figures 3B,C). Considering the sex- and strain-specific effects of VitD on EAE (see Figure 2), and the profound effect of genotype, we analyzed the effect of VitD in each of the four sex/strain combinations (B6 females, B6 males, PWD females, and PWD males) separately. Two major findings were noted. First, in Teff cells, a prominent effect of VitD on gene expression was detected in B6 females, which was absent in B6 males or PWD mice of either sex (Figure 3B), in concordance with the effect of VitD on EAE outcome (see Figure 2). Second, in Tregs, VitD exhibited the strongest effect in PWD females (Figure 3C). In both cases, high VitD predominantly induced upregulation of gene expression compared with low VitD (Figures 3B,C).

Bioinformatic pathway analyses of DE genes (see Materials and Methods) revealed divergent and sometimes opposing effects of VitD on gene expression, depending on sex and strain (Figure 3D). Several canonical pathways relevant to EAE/MS pathogenesis (including neuroinflammation, Th1, and a number of other proinflammatory pathways) were downregulated by high VitD in Teff cells from B6 females, yet upregulated in B6 males or PWD males. The gene expression patterns in Teff cells were again concordant with EAE outcomes, whereby downregulation of proinflammatory activity by VitD was associated with EAE suppression in B6 females. Similarly, upstream regulator analysis in B6 female Teff cells predicted significant inhibition of several proinflammatory nodes by high VitD, including TNF, NFκB, CSF2, and IFNγ as the top four nodes. Comparison of DE transcripts within the IFNγ node with two selected MS relevant canonical pathways, “Neuroinflammation Signaling Pathway” (ranked first) and “Th1 Pathway” (ranked 11th) revealed strong functional overlap and several key shared transcripts, including several MHC class II genes, and a central role for IFNγ itself (Figure S2 in Supplementary Material). The former is consistent with the documented regulation of the major MS risk MHC class II allele, HLA-DRB1*15:01, by VitD. The latter is consistent with the documented critical role of IFNγ in the regulation of EAE by VitD (36). Finally, this analysis implicated *Mapk14* (Figure S2 in Supplementary Material), encoding p38α MAP kinase, a gene that we have previously identified as a central regulator of differential sex-specific genetic effects on EAE in the B6.Chr^PWD^ model (51), a hypothesis that was validated in our targeted analysis of the role of p38α in EAE (52).

To test whether the observed regulation of immune cell transcriptomes by VitD had direct connections to mechanisms of CNS autoimmunity in humans, we compared the level of enrichment of VitD-dependent DE genes in PWD cells within the set of transcripts that were reported to be upregulated or downregulated in CD4 T cells isolated from early onset MS patients (clinically isolated syndrome; MS-CIS) relative to healthy controls (MS-CIS signature genes) (53). VitD-dependent DE genes in Teff cells from B6 females exhibited highly significant enrichment (*p* = 1.02e−6) within genes *downregulated* in MS-CIS CD4 cells, while marginal or no enrichment was observed for genes that were *upregulated* in MS-CIS (Figure 3E). Marginal to no enrichment was observed in either direction for other strain-sex combinations in Teff cells (Figure 3E) or for any of the DE gene sets in Tregs. Together with the observation that the majority of DE genes in B6 female Teff cells are *upregulated* with high VitD (Figure 3B), these data suggest that high VitD normalizes the expression of genes that are downregulated in MS-CIS CD4 T cells, which are likely associated with MS pathogenesis. Taken together, our results indicate that high VitD suppresses MS-associated proinflammatory gene expression programs in CD4 T cells in a sex-, cell type-, and genotype-specific manner, in concordance with its protective effects on EAE.

### Sex and Genotype Dictate the Outcome of VitD Modulation in EAE

**Figure 4:**
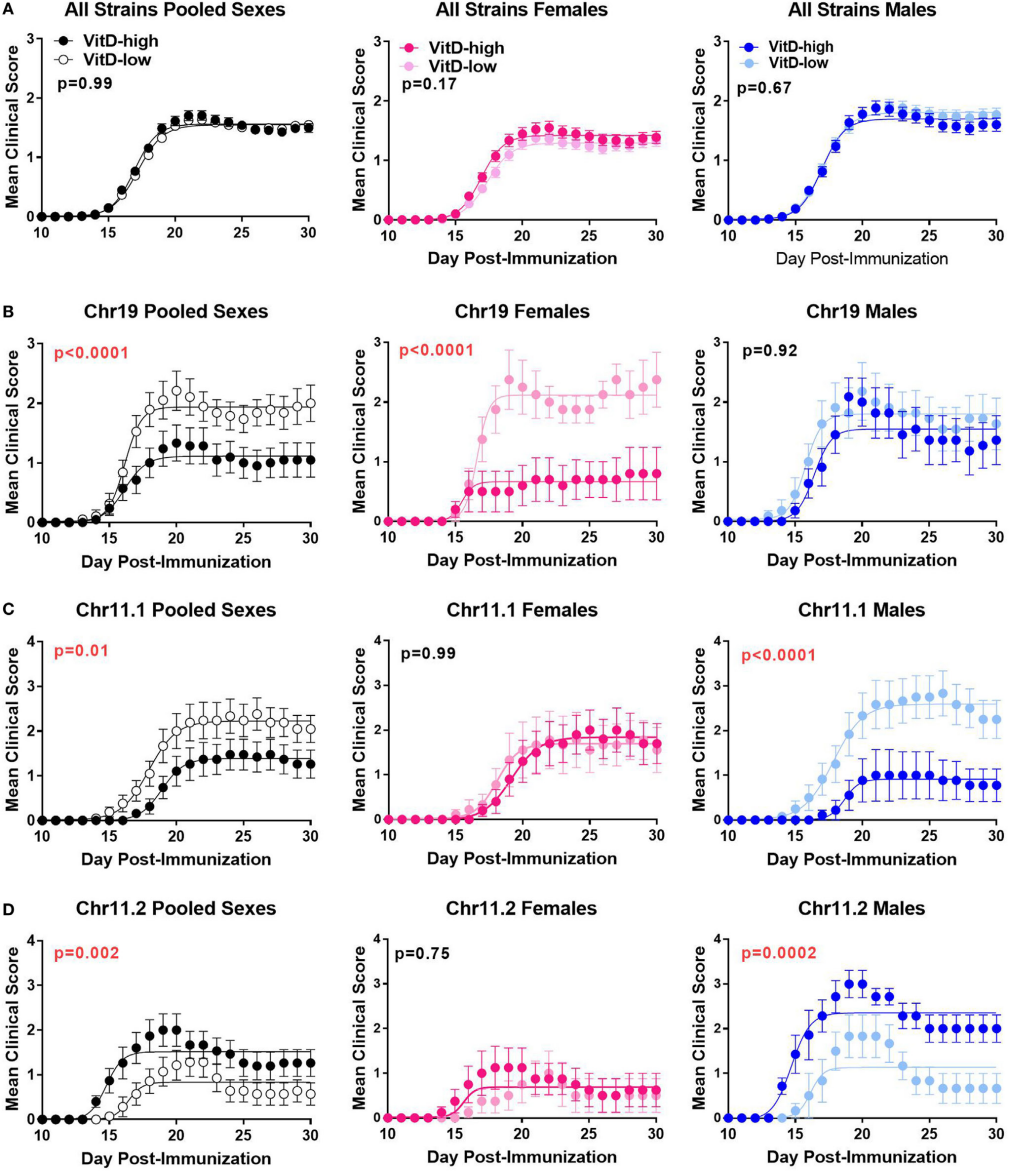
Effects of vitamin D3 (VitD) on EAE disease course in B6.Chr^PWD^ consomic mice. B6.Chr^PWD^ consomic mice were exposed to VitD-high and VitD-low diets, as in Figure 2, followed by induction of EAE. Disease course in a pooled analysis of all consomic strains **(A)**, or in three representative consomic strains, Chr19^PWD^ **(B)**, Chr11.1^PWD^ **(C)**, and Chr11.2^PWD^ **(D)**, is shown for pooled sexes, or for each sex separately, as indicated. *p*-Values for the effect of diet on overall EAE course (representing the interaction term for treatment × time, repeated measures two-way ANOVA) are shown. Numbers of animals per group are shown in Table S1 in Supplementary Material.

Since epidemiologic data (6, 26–29) and our findings above suggest the possibility that the outcome of VitD status in MS and EAE may be dependent on genotype, we deliberately introduced the segregation of natural genetic variation into our model. To achieve this, we utilized the B6.Chr^PWD^ consomic model, in which natural genetic variation exhibits a significant impact on EAE susceptibility (51). Twenty B6.Chr^PWD^ consomic strains, encompassing 17 autosomes, X and Y, and a conplastic strain with the PWD mitochondrial genome were included in the study. The mice were exposed to the VitD-high and VitD-low dietary paradigm, followed by EAE induction and evaluation, as in Figure 2. Surprisingly, in a combined analysis of all strains and sexes, no significant effect of dietary modulation of VitD on EAE course was detected (Table 1; Figure 4A). Similarly, when this combined analysis was stratified by sex, no significant effect of VitD in either sex was detected (Table 1; Figure 4A). Given the known variation in EAE susceptibility across the B6.Chr^PWD^ consomic/conplastic strains (51), and the sex- and strain-specific responses to VitD in EAE and gene expression (see above), each strain was analyzed individually for the effect of VitD on EAE, either combining both sexes or analyzing each sex separately. When analyzing males and females together, five strains showed significant effects of VitD. Chr11.1^PWD^ and Chr19^PWD^ had lower EAE severity on VitD-high diet compared with VitD-low, as seen in B6 females, but surprisingly, Chr10.2^PWD^, Chr11.2^PWD^, and Chr14^PWD^ exhibited the opposite effect (Figure 4; Figure S3 in Supplementary Material). Stratification by sex revealed that these effects were primarily driven by a single sex, either female or male, depending on the strain (e.g., Figure 4). Stratification by sex also revealed additional significant effects of VitD in females (Chr10.3^PWD^, Chr15^PWD^, and mt^PWD^) and in males (Chr8^PWD^, Chr12^PWD^, and ChrY^PWD^) (Table 1). Additional analyses of EAE quantitative trait variables (Table S1 in Supplementary Material) supported the conclusions drawn from disease course analyses (Table 1; Figure 4; Figure S4 in Supplementary Material).

**Table 1:** Effects of vitamin D3 (VitD) on EAE disease course in B6.Chr^PWD^ consomic mice.

*N*/group			Effect of diet on EAE course^a^		
Strain	Female	Male	Pooled sexes	Females	Males
All strains	449	448	ns	ns	ns
B6	52	36	ns	0.006 ↓	ns
PWD	10	13	ns	ns	ns
Chr1	21	18	ns	ns	ns
Chr2	6	6	ns	ns	ns
Chr5	21	24	ns	ns	ns
Chr6	30	21	ns	ns	ns
Chr7	11	22	ns	ns	ns
Chr8	9	10	ns	ns	0.006 ↑
Chr9	22	19	ns	ns	ns
Chr10.2	14	20	0.002 ↑	ns	0.006 ↑
Chr10.3	25	24	ns	<0.0001 ↑	ns
Chr11.1	19	21	0.01 ↓	ns	<0.0001 ↓
Chr11.2	16	13	0.002 ↑	ns	0.002 ↑
Chr12	29	29	ns	ns	0.0001 ↓
Chr14	18	22	<0.0001 ↓	ns	<0.0001 ↓
Chr15	20	23	ns	0.03 ↓	ns
Chr17	19	19	ns	ns	ns
Chr18	26	25	ns	ns	ns
Chr19	18	22	<0.0001 ↓	<0.0001 ↓	ns
ChrY	27	26	ns	ns	<0.0001 ↓
ChrX.3	11	12	ns	ns	ns
mt	25	23	ns	<0.0001 ↑	ns

*^a^The significance of the observed differences in the severity of the EAE clinical disease course for each of the B6.Chr^PWD^ consomic strains was analyzed for the effect of VitD diet, as described in Section “Materials and Methods.” Data for females and males were analyzed together (pooled) and following stratification by sex. ANOVA *p*-values for the effect of diet on overall EAE course are shown where significant; ns, not significant; ND, not done. Direction of arrow indicates the direction of change for EAE severity in the VitD-high diet group relative to the VitD-low group (i.e., downward arrow indicates suppression of EAE by VitD-high diet)*.

### Systemic 25(OH)D Levels Predict EAE Outcome in Female B6.Chr^PWD^ Consomic Mice

**Figure 5:**
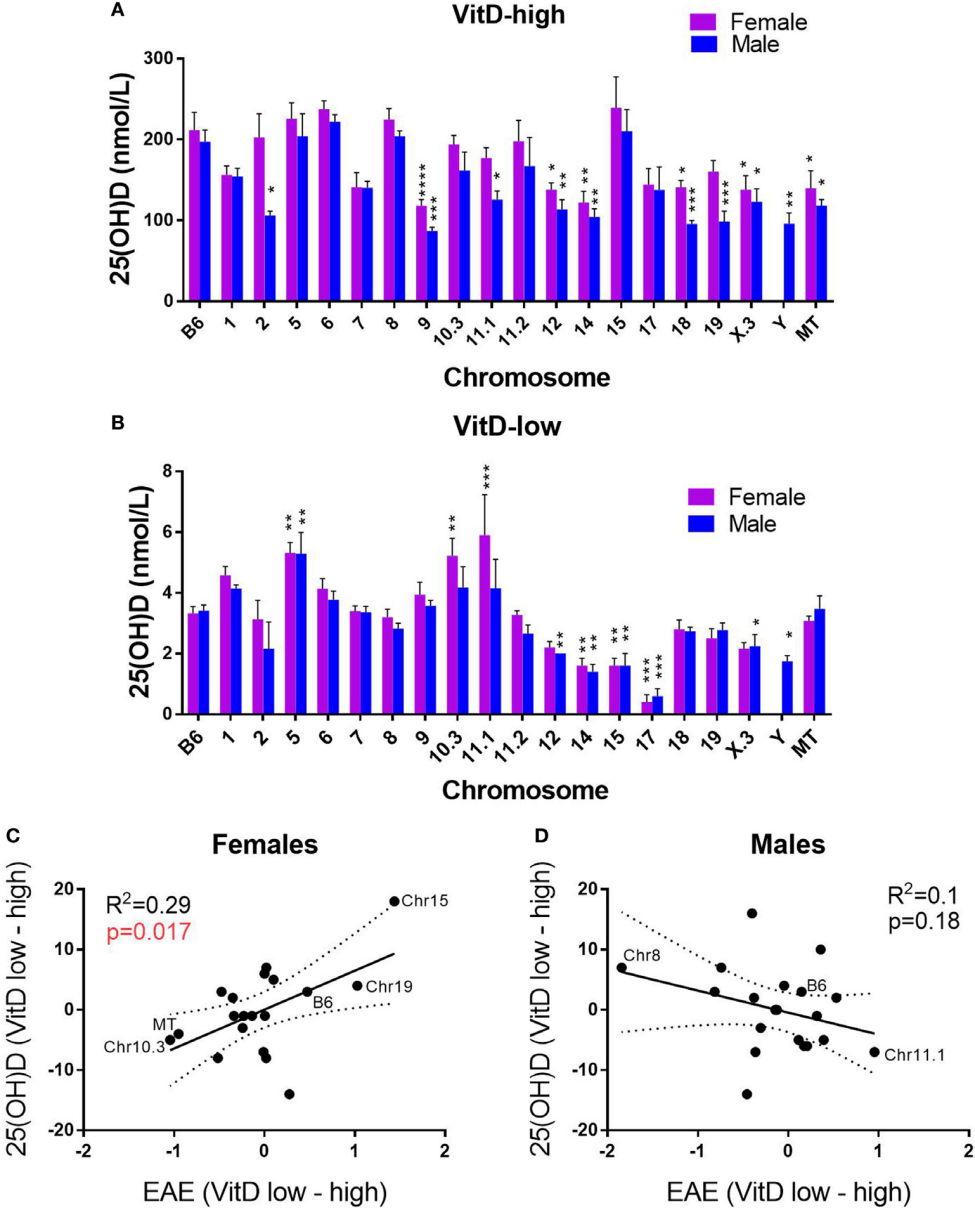
Effects of genotype and sex on systemic vitamin D3 (VitD) levels as related to EAE outcomes. B6.Chr^PWD^ consomic mice were exposed to VitD-high **(A)** and VitD-low **(B)** diet, followed by EAE induction, as in Figure 4. Sera were collected at day 30 post-EAE induction. 25(OH)D levels were measured by enzyme-linked immunoassay in five males and five females of each strain for each diet type. The significance of the observed differences was assessed by two-way ANOVA, followed by Holm–Sidak’s *post hoc* comparisons, comparing each consomic strain to B6. Symbols indicate a significant difference between B6 and the indicated groups, as follows: *p < 0.05; **p < 0.01; ***p < 0.001; ****p < 0.0001. **(C,D)** The relationship between serum 25(OH)D levels and EAE severity was examined, as described in Section “Materials and Methods.” Linear regression was used to determine the significance of the correlation between the ranked 25(OH)D response [difference in 25(OH)D between VitD-low and -high diets] and normalized EAE response (difference in cumulative disease score between VitD-low and high diets). *R*^2^ values and *p*-values for regression analysis are shown. Selected B6.Chr^PWD^ consomic strains are labeled as specific examples.

Since circulating VitD levels are genetically controlled in humans (54) and in B6 and PWD mice (Figure 1), we determined whether B6.Chr^PWD^ consomic/conplastic strains subjected to our dietary paradigm exhibited any differences in systemic VitD levels. Because the 25(OH)D levels reached on the VitD-high diet were dramatically different from those on the VitD-low diet (see Figure 1), the data were analyzed separately for each diet, to assess the effect of strain and sex. Significant differences in 25(OH)D levels were observed between several consomic strains and B6. On the VitD-high diet, a number of strains presented with significantly lower levels of 25(OH)D3 compared with B6, but no strains exhibited higher levels (Figure 5A). On the VitD-low diet, several strains exhibited significantly higher or lower levels of 25(OH)D compared with B6 (Figure 5B). Next, we tested whether any of these changes in 25(OH)D levels were correlated with EAE outcomes, e.g., whether a larger change in VitD levels corresponded to a higher degree of disease protection by VitD. EAE cumulative disease score (CDS), as the quantitative trait variable that most accurately reflects the overall severity of the EAE clinical disease course, was used to calculate a normalized difference in CDS between VitD-low and -high diets, for each strain. Similarly, a relative difference in serum 25(OH)D between VitD-low and -high diets was calculated for each strain. The relationship between these two parameters was examined using linear regression. For female consomic mice, a significant positive relationship was observed, suggesting that those strains that had higher serum 25(OH)D responses exhibited protective effects of VitD (e.g., B6, Chr15, and Chr19), while those with low responses had the opposite effects (e.g., Chr10.3), albeit the association was modest (*r*^2^ = 0.29) (Figure 5C). Surprisingly, in males, the trend was reversed, although it did not reach significance (Figure 5D). Taken together, these results suggest that genotype controls systemic levels of VitD, which in turn may contribute to EAE susceptibility in a sex-specific manner.

### Tissue-Specific Differential Expression of VitD Metabolic Pathway Genes in B6 and PWD Mice

**Figure 6:**
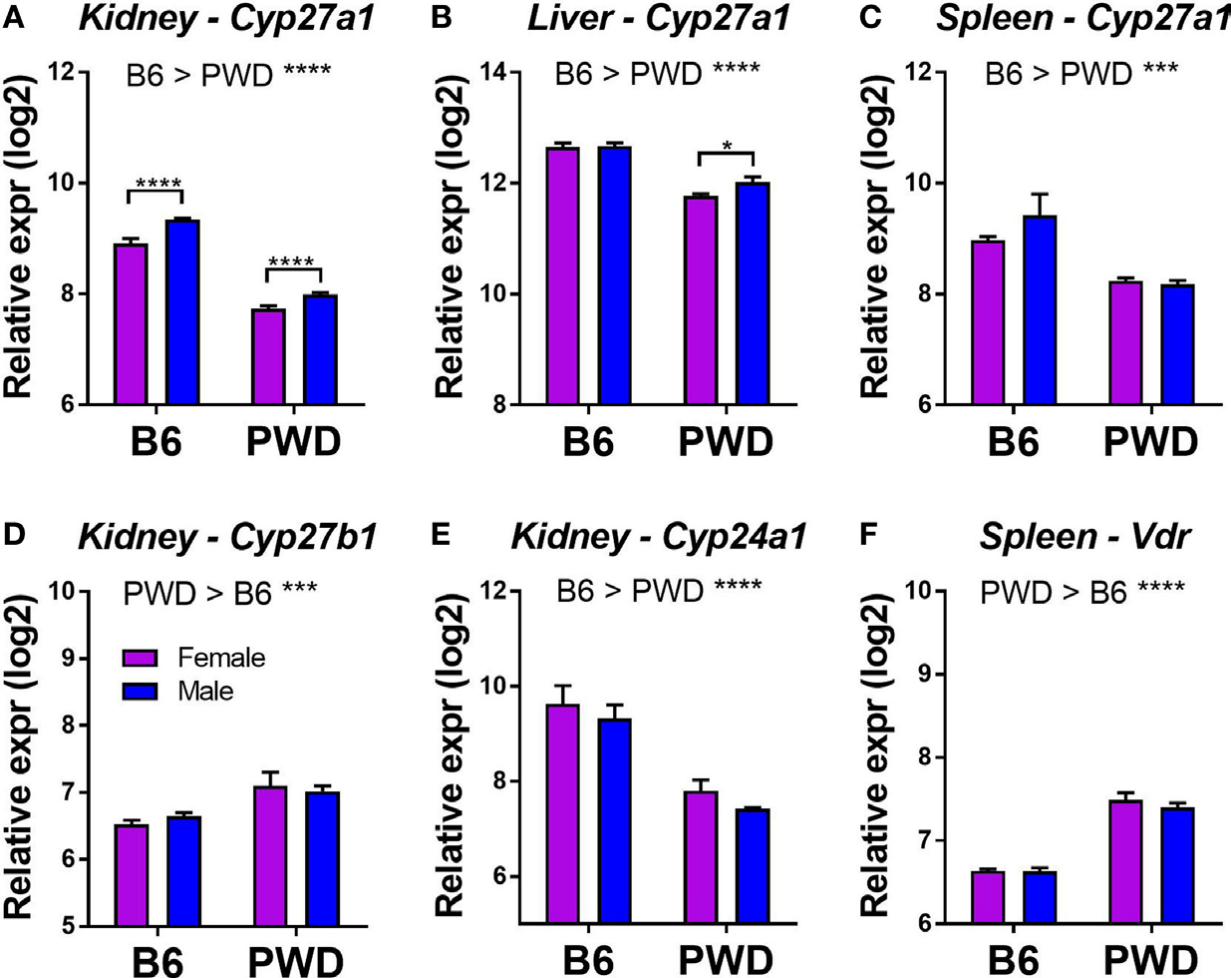
Tissue-specific differential expression of vitamin D3 metabolic pathway genes in B6 and PWD mice. Gene expression data were obtained from the Gene Expression Miner database, as described in Section “Materials and Methods.” Genes showing a significant differential expression (with a greater than 30% change in expression) as a function of strain or sex are shown in **(A–F)**. The significance of the observed differences was assessed by two-way ANOVA, with Holm–Sidak’s *post hoc* comparisons: B6 vs. PWD (overall effect of strain; indicated above the graphs), and female vs. male (within strain; indicated with brackets). The data included 12 C57BL/6J mice (6 females and 6 males), and 11 PWD/PhJ mice (6 females and 5 males). Symbols indicate a significant difference between the indicated groups, as follows: *p < 0.05; **p < 0.01; ***p < 0.001; ****p < 0.0001.

Systemic 25(OH)D levels in humans are genetically regulated, and recent GWAS studies have identified common variants in four major genes in the VitD metabolism pathway: *GC, DHCR7, CYP2R1*, and *CYP24A1*, which together explain a large proportion of the variation in 25(OH)D (54). Subsequent Mendelian randomization studies showed that the genetic control of low 25(OH)D levels by alleles of the four genes is associated with increased susceptibility to MS (11–13). To begin to identify potential candidate genes underlying differential responses across different B6.Chr^PWD^ consomic strains, we examined the level of expression of mouse orthologs of these four key genes (*Gc, Dhcr7, Cyp2r1*, and *Cyp24a1*) in several relevant tissues (kidney, liver, and spleen) in male and female B6 and PWD mice, using publically available datasets (see Materials and Methods). We included three additional key VitD metabolism genes: *Cyp27a1, Cyp27b1*, and *Vdr* (55). A number of these genes were DE across different tissues. In all three tissues, vitamin D-25 hydroxylase, *Cyp27a1*, exhibited twofold to threefold lower expression in PWD compared with B6, with significantly lower expression in females compared with males in both strains in the kidney (Figures 6A–C). In the kidney, 1-α-hydroxylase, *Cyp27b1*, exhibited a modest significant increase in expression in PWD compared with B6 (Figure 6D), while 1,25(OH)_2_D_3_-inactivating 24-hydroxylase, *Cyp24a1*, exhibited a significant increase in expression in B6 (Figure 6E). *Vdr* also exhibited higher expression in PWD compared with B6 in the spleen (Figure 6F). These results demonstrate genetic control of tissue-specific differential expression of several components of the VitD metabolic pathway which may underlie some of the differences in serum 25(OH)D levels (Figures 1 and 5) and EAE responses (Table 1; Figure 4) in B6.Chr^PWD^ mice. Of these genes, two lie within consomic intervals of interest (carried by consomic strains exhibiting significant effects in Table 1): *Cyp27b1* (Chr10.3) and *Vdr* (Chr15). As such, they represent candidate genes controlling differential responsiveness to VitD in EAE (see Discussion), and their relevance will be assessed in future studies.

## Discussion

Genetic and environmental influences on MS risk are well documented. However, gene–environment interactions have been more elusive, with the exception of HLA alleles and their putative interactions with smoking, EBV, and obesity (56). Part of the difficulty in identifying such interactions is the inability to clearly define over time the presence/absence/level of environmental variables/exposures and possible confounding variables (57). The other difficulty inherent to all epidemiologic studies is the inability to separate cause and effect from association/correlation. The association of low VitD with MS risk is an example of such an association, where cause and effect have been difficult to parse out, and therapeutic intervention has not yet provided a clear answer (58). Animal models provide an opportunity to bridge the gap between observation and causation, as putative genetic and environmental risk factors can be precisely controlled (45). As such, they also provide an opportunity to identify gene–environment interactions in a highly controlled experimental setting. In this study, we have applied a well-defined dietary paradigm to intentionally modulate systemic VitD levels, while at the same time introducing controlled genetic variation. This approach revealed that the effects of VitD in a mouse model of MS are regulated by sex and genotype in a cell type-specific fashion.

In a seminal prospective case–control study, Ascherio and colleagues identified an association between low 25(OH)D levels and increased risk of MS in a white U.S. population (6). Interestingly, in the same study, this association was absent in black and Hispanic groups, who in fact had lower 25(OH)D levels compared with whites. This lack of association between 25(OH)D and MS in non-whites was confirmed by several groups (22, 26–29), which has led to the suggestion that VitD-related testing and treatment in MS should be informed by ethnicity (59). In our study, we provide complementary experimental evidence that genotype can influence the outcome of VitD status or supplementation. This may help explain the variability in VitD supplementation trials in MS so far, and our results also suggest that such studies might benefit from a complementary pharmacogenetic approach to identify gene variants associated with positive or negative responses to VitD.

In this regard, there is evidence that common genetic variants in *GC* (encoding the vitamin D binding-protein; DBP) may lead to profound ethnic-specific variations in DBP levels, DBP binding avidity, and bioavailablity of VitD and its metabolites (31). However, a recent study by Barcellos and colleagues found that these variants in *GC* do not account for the lack of association between serum VitD levels and MS in blacks and Hispanics (30). This suggests that other unknown ethnic-specific genetic determinants can regulate VitD metabolism and/or subsequent physiologic responses, such as those underlying MS susceptibility.

In some B6.Chr^PWD^ consomic strains (e.g., male Chr11.2^PWD^), an unexpected phenotype was observed, whereby higher EAE severity was seen on the VitD-high diet compared with VitD-low diet. However, several previous reports have documented similar unexpected EAE-suppressing effects of VitD insufficiency or deficiency, depending on length and timing of the exposure (60, 61). In addition, mice completely deficient in *Vdr* are unexpectedly resistant to EAE (62), suggesting that VitD signaling is needed to mount a robust T cell response. Thus, we hypothesize that VitD status can serve as a bi-directional rheostat regulating autoimmunity, and this balance can be further modified by genetic background and sex. Interestingly, Chr10.3^PWD^ female mice exhibited lower 25(OH)D responses associated with increased EAE on the VitD-high diet (Figure 5C; Figure S3 in Supplementary Material). It is possible that increased kidney *Cyp27b1* expression from the PWD allele in the Chr10.3 locus (Figure 6D) drives faster 25(OH)D to 1,25(OH)D metabolism, which could result in different levels or kinetics of VDR activation and divergent EAE outcomes. Conversely, female Chr15^PWD^ mice exhibit the opposite phenotype with regard to VitD status and EAE outcome (Figure 5C), while the PWD allele of *Vdr* (located on Chr15) shows higher expression (Figure 6F). Since for *Cyp27b1* and *Vdr* the expression differences were not sex specific (Figures 6D,F), we postulate that other sex-specific factors (e.g., lower expression of *Cyp27a1* in females; Figures 6A,B) interact with the genetically determined differential expression of these VitD pathway genes to give rise to different outcomes in EAE.

In a recent study, Jagodic and colleagues used a dietary paradigm highly similar to ours to examine genomic effects of VitD in the inbred Dark Agouti rat model of EAE (44). The conclusions reached by the authors were largely similar to ours, whereby supplementation of female rats with high levels of VitD induced an anti-inflammatory gene expression program in CD4 Teff cells from immunized mice, in concordance with amelioration of EAE. Comparison of the DE gene set from this study with our DE gene set did not reveal strong gene–gene correlation, suggesting species differences, differences in cell isolation protocols, and/or timing of cell isolation. Nonetheless, many of the differentially regulated pathways showed strong agreement between the two studies (e.g., MAPK signaling, NFκB, etc.), suggesting that a similar immune-regulatory phenotype may be achieved by VitD in both situations.

Despite the strong epidemiological associations, the role of VitD in MS remains complex and unclear. Our studies add another layer to this complexity: the immunologic response to VitD status may differ across individuals due to genetic and sex effects. The sex effects have been shown in mouse models to be likely due to the influence of estrogen (42). Our future studies will be aimed at identifying genetic modifiers of the VitD response in CNS autoimmunity. This information will help inform future VitD supplementation trials, as well as the use of VitD status as a prognostic.

## Materials and Methods

### Animals and Dietary Treatments

C57BL/6J (B6), PWD/PhJ (PWD), and B6.Chr^PWD^ consomic mice were purchased from Jackson Laboratories (Bar Harbor, ME, USA), then bred and housed in a single room within the vivarium at the Larner College of Medicine at the University of Vermont for two to four generations prior to any experimentation. The experimental procedures used in this study were approved by the Animal Care and Use Committee of the University of Vermont.

To ensure their correct identity and to enhance rigor and reproducibility of these studies, B6.Chr^PWD^ consomic mice were subjected to genome-wide SNP genotyping using Dartmouse genotyping services (Dartmouth College, NH, USA). All mice used in this study were of the expected genotypes, with the following exceptions. B6.Chr4^PWD^ mice were excluded from the study, because they were found to be a mix of various genotypes, where much of the Chr4^PWD^ had been replaced with B6 genome. Chr17^PWD^ mice were found to carry a homozygous B6-derived interval between 30 and 45 Mb on Chr17, encompassing *H2*.

At weaning (3 weeks of age), littermate mice were randomly assigned to one of two diets: VitD-low (0 IU VitD/g; 0.87% Ca^2+^, 0.3% phosphorus, alcohol extracted casein as protein source) or VitD-high [identical composition to VitD-low, supplemented with 5 IU VitD (cholecalciferol)/g]. The composition of the diets was based on the study those described by Spach and Hayes (41) and was prepared by Harlan-Teklad (WI, USA), designated by the following company product numbers: VitD-low, TD.10837; VitD-high, TD.140867.

### Induction and Evaluation of EAE

EAE was induced in B6 and B6.Chr^PWD^ consomic mice using the 2× MOG35-55/CFA protocol, as previously described (63). Mice were injected subcutaneously with 0.1 ml of emulsion containing 0.1 mg of myelin oligodendrocyte glycoprotein peptide 35–55 (MOG_35–55_) peptide (Anaspec Inc., MA, USA) in PBS and 50% complete Freund’s adjuvant (CFA; Sigma, USA) on day 0 on the lower flanks (50 µl per flank), followed by an identical injection on upper flanks on day 7. CFA was supplemented with 4 mg/ml *Mycobacterium tuberculosis* H37Ra (Difco, USA). EAE was induced in PWD mice using the following modifications to the protocol above, as previously described (50). Mice were injected subcutaneously with 0.1 ml of emulsion containing 2.5 mg of MSCH in PBS and 50% CFA on day 0 and day 7. On day 0 and day 2, mice also received an i.p. injection of 200 ng pertussis toxin (List Laboratories, USA) as an ancillary adjuvant.

Starting on day 10, mice were scored visually, as follows: 1—partial loss of tail tone, 2—full loss of tail tone, 3—loss of tail tone and weakened hind limbs, 4—hind limb paralysis, 5—hind limb paralysis and incontinence, and 6—quadriplegia or death. EAE scoring was performed by a non-biased observer. EAE quantitative traits were calculated essentially as previously described (64), as follows. The incidence of EAE was recorded as positive for any mouse with clinical signs of EAE (clinical score ≥1) for two or more consecutive days. CDS was calculated as the sum of all daily scores over the course of 30 days. Days affected was calculated as the number of days an animal displayed a clinical score ≥1 for at least two consecutive days. Day of onset was the day a clinical score ≥1 was first observed (not calculated for animals without clinical signs for at least two consecutive days). Severity index was generated by averaging the clinical scores for each animal over the number of days that it exhibited clinical symptoms (unaffected animals were included as 0). Peak score represents the maximum daily score (unaffected animals were included as 0).

### Cell Sorting and RNA Isolation

B6 and PWD mice were subjected to the dietary paradigms, as described in Section “Results.” At 5 weeks post-dietary intervention, mice were euthanized, and spleens were collected. Spleens were digested using Spleen Dissociation Medium (STEMCELL Technologies, Inc., Canada). B cells were depleted using the EasySep B cell positive selection kit and EasySep magnet (STEMCELL Technologies, Inc., Canada). The remaining cells were purified by FACS using fluorophore-conjugated antibodies against cell surface markers as follows: CD4 (Tconv) cells (CD19^−^ TCRβ^+^ CD4^+^ CD8^−^ CD25^−^) and Tregs (CD19^−^ TCRβ^+^ CD4^+^ CD8^−^ CD25^+^). Dead cells were excluded using the Far Red Live-Dead staining kit (Thermo Fisher Scientific, USA). Antibodies were purchased from BioLegend, Inc. (San Diego, CA, USA); catalog numbers were as follows: CD19, CD4, CD25, CD8, CD11b, CD11c, and TCRβ; 115534, 100531, 102016, 101206, 117319, and 109222, respectively. RNA was isolated using the Qiagen RNeasy Mini or Micro Kits. RNA quality was assessed using the Agilent Bioanalyzer 2100, and samples were selected for downstream analysis based on RNA integrity number (typically 6–9). RNA quantity was determined using Qubit Fluorometric Quantification (Thermo Fisher Scientific, USA). Four biological replicates (individual mice) for each strain, sex, and diet combination were selected.

### Transcriptional Profiling

For transcriptional profiling, microarray analysis was performed at the UVM Cancer Center Genomics Facility using the Mouse Affymetrix Clariom D Genechip and the GeneChip™ WT Pico Target Preparation reagent kit (Thermo Fisher Scientific 9026220) as described by the manufactures procedures. Briefly, 5 ng of RNA was used to synthesize cDNA through a First-Strand and Second-Strand reverse transcription reaction followed by conversion to cRNA through an overnight T7 InvitroTranscription reaction. The resulting cRNA was purified and 5.5 μg was converted to sense, single-strand cDNA using UDG (10 U/μL) and APE1 (1,000 U/μL), provided in the GeneChip^®^ WT PLUS Reagent Kit. cDNA was end labeled with biotin using TdT (30 U/μL), and used as input for the hybridization mix for the GeneChip. Mouse Clariom D arrays were incubated in the Affymetrix^®^ GeneChip^®^ Hybridization Oven 645 at 45°C/60 RPM for 16–18 h. Arrays were stained using the Affymetrix^®^ GeneChip^®^ staining reagents and scanned with the 7 G Affymetrix^®^ GeneChip^®^ Scanner 3000.

### Statistical Analyses of Microarray Data

Raw intensity CEL files were imported into Expression Console software (Affymetrix, USA), and CHP files were generated for gene level analysis. CHP files were imported into Transcriptome Analysis Console (TAC) software v4.0.0.25 (Affymetrix, USA), and gene level expression analysis was performed using the default ANOVA settings (e-Bayesian method), analyzing Tconv and Treg data separately. The following comparison variables were used: strain (B6, PWD), sex (male, female), diet (VitD-high, VitD-low), as well as a batch variable (cDNA samples were processed and scanned in batches over several different days). To detect DE genes as a function of VitD status, pairwise comparisons were done between VitD-high and VitD-low groups for the following sample groupings: (all mice together, B6 females only, B6 males only, PWD females only, and PWD males only). All raw microarray data have been deposited into the Gene Expression Omnibus database, accession number GSE116457.

### Bioinformatic Analyses

Pathway analysis was performed using Ingenuity Pathway Analysis™ (IPA; Qiagen, Inc., USA) software. The gene expression datasets were exported from TAC software and uploaded into IPA. A relaxed cutoff filter of |FC| > 1.5 and ANOVA *p* < 0.05 was used to maximize the number of genes in the analysis (recommended by IPA to enhance the analysis power and accuracy). The IPA Core Analysis function, followed by the Comparison Analysis function was used to compare the effect of VitD across the four strain-sex combinations (B6 females, B6 males, PWD females, and PWD male), as follows. The Canonical Pathway function was used to identify the top canonical pathways (*p* < 0.01, *Z*-score > |2|) affected by the DE genes between VitD-high and VitD-low conditions. The sign and magnitude of the *Z*-scores are indicative of the predicted strength and direction of the VitD-high effect. The upstream regulator analysis function was similarly used to predict *Z*-scores and *p*-values for putative upstream regulators.

Enrichment analysis of VitD-regulated genes compared with genes DE in CD4 T cells in MS as performed as follows. The list of transcripts reported to be upregulated in CD4 T cells from MS-CIS subjects vs. controls (53) was imported into IPA. The Core Analysis function was used to determine the significance of enrichment of VitD DE genes within the MS-CIS list.

### Serum 25(OH)D and Ca^2+^ Measurements

Whole blood was collected at the indicated time points, allowed to coagulate for 30 min at room temperature, followed by centrifugation and collection of serum. Sera were stored at −80°C prior to analysis. 25(OH)D levels were determined using a commercially available enzyme-linked immunoassay kit (ImmunoDiagnosticSystems, Inc., MD, USA), which detects 25(OH)D_3_ and 25(OH)D_2_. Sera from mice on the high VitD diet were diluted 1:10 with PBS + 1% BSA prior to analysis, to maintain readings within the range of the standard curve. Kit-supplied standards were used with 4-parameter logistic regression to determine concentrations in unknown samples.

Serum Ca^2+^ measurements were performed at the University of Vermont Medical Center Clinical Chemistry laboratory.

### Statistical Analyses

Statistical analyses not pertaining to microarray data were carried out using GraphPad Prism software, version 6. Details of the analyses are provided in the figure legends and below. All statistical tests were two-sided, and adjustments for multiple comparisons were made as indicated. All center values represent the mean, and error bars represent the SEM. *p*-Values below 0.05 were considered significant. Sample sizes for animal experiments were chosen based on previous experience with similar analyses. Animals were randomly assigned to different treatment groups (diet), assigning littermates to both groups evenly, whenever possible. For some consomic strains, sample sizes studied were lower due to inadequate breeding performance.

Analyses of EAE clinical scores were performed as follows. Clinical disease time course was analyzed for the effect of diet (VitD) for each strain, using two-way repeated measures ANOVA. The effect of diet was considered significant when a significant strain and/or strain × time interaction term was observed. The latter term is shown in the figures and tables, to indicate overall significance of effect of diet on overall EAE course.

The relationship between serum 25(OH)D levels and EAE severity was determined as follows. First, a weighted difference in EAE severity was calculated, using CDS, as a single quantitative measure of overall disease severity and duration. For each strain, the diet-driven change in CDS was calculated by substracting the mean CDS for the VitD-high diet from the mean CDS for the VitD-low diet. Since the absolute CDS varied significantly across strains independent of VitD, this change was normalized by dividing by the overall mean CDS for that strain:

$$\text{Normalized change in EAE CDS}=\frac{{\text{CDS}}_{\text{VitD}-\text{low}}-{\text{CDS}}_{\text{VitD}-\text{high}}}{{\text{mean CDS}}_{\text{VitD}-\text{low}+\text{high}}}.$$

Because serum 25(OH)D levels varied by strain, to determine the relative serum 25(OH)D response to diet, a ranked change was calculated for each strain, as follows. 25(OH)D3 levels were ranked for across the consomic strains separately for VitD-high and VitD-low diets. For each strain, the ranked change in 25(OH)D as a function of diet was calculated by substracting the ranked 25(OH)D for the VitD-high diet from the ranked 25(OH)D for the VitD-low diet:

Ranked change in 25(OH)D =ranked 25(OH)DVitD−low−ranked 25(OH)DVitD−high.

Subsequently, linear regression was used to determine measure the association between the ranked 25(OH)D3 response and normalized EAE response. *R*^2^ and the significance for the slope not being equal to zero were calculated as a measure of strength of association.

### Differential Expression Analyses of VitD-Related Genes in B6 and PWD Mice

Differential expression data were obtained from the Gene Expression Miner database from Jackson Laboratories (http://cgd.jax.org/gem/strainsurvey26/v1), which contains microarray data from a gene expression survey across 26 inbred strains of mice, in 4 tissues: spleen, liver, and left and right kidneys. The data set included data on 12 C57BL/6J mice (6 females and 6 males), and 11 PWD/PhJ mice (6 females and 5 males). Log2-normalized gene expression data were downloaded for the following genes of interest: *Gc, Dhcr7, Cyp2r1, Cyp24a1, Cyp27a1, Cyp27b1*, and *Vdr*. *Gc* and *Cyp2r1* did not show appreciable levels of expression in any of the tissues, and thus were not analyzed further. The rest of the gene expression data were analyzed in Graphpad Prism, version 6, as detailed in the figure legends.

## Ethics Statement

This study was carried out in accordance with the recommendations of AAALAC guidelines. The protocol was approved by the Animal Care and Use Committee of the University of Vermont.

## Author Contributions

DK and CT designed experiments. DK, LA, and CT analyzed data and wrote manuscript. DK, QF, and MM performed experiments and analyzed data.

## Conflict of Interest Statement

The authors declare that the research was conducted in the absence of any commercial or financial relationships that could be construed as a potential conflict of interest. The reviewer FL and the handling Editor declared their shared university affiliation and declare that they are working in different departments.
