# Supplementary material for: Sex-Specific Gene-by-Vitamin D Interactions Regulate Susceptibility to Central Nervous System Autoimmunity
Source: Front Immunol. 2018 Jul 17;9:1622. doi: 10.3389/fimmu.2018.01622 (PMC6056725; doi:10.3389/fimmu.2018.01622)
Supplement: Supplementary file 1 [file data_sheet_1.docx]

Supplementary Material

**Sex-specific gene-by-vitamin D interactions regulate**

**susceptibility to CNS autoimmunity**

**Dimitry N. Krementsov^1, *^, Loredana Asarian^2^, Qian Fang^2^, Mahalia M. McGill^1^, Cory Teuscher^2, 3, *^**

**^*^ Address correspondence to:**

Dimitry Krementsov, PhD, [dkrement@uvm.edu](mailto:dkrement@uvm.edu)

**Supplemental Figures**


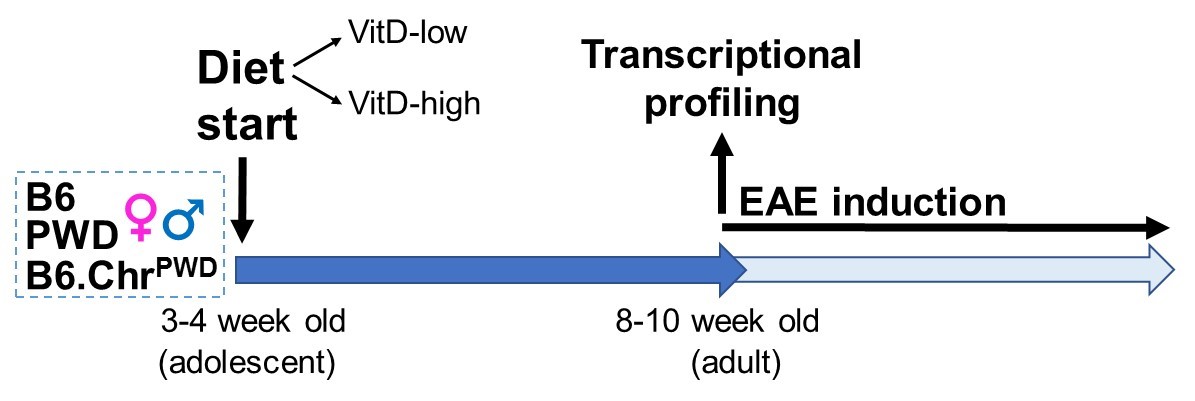


**Supplemental Figure 1. Schematic overview of the experimental design.** Dietary regimens were initiated at 3-4 weeks of age and maintained until the end of experimentation.

**
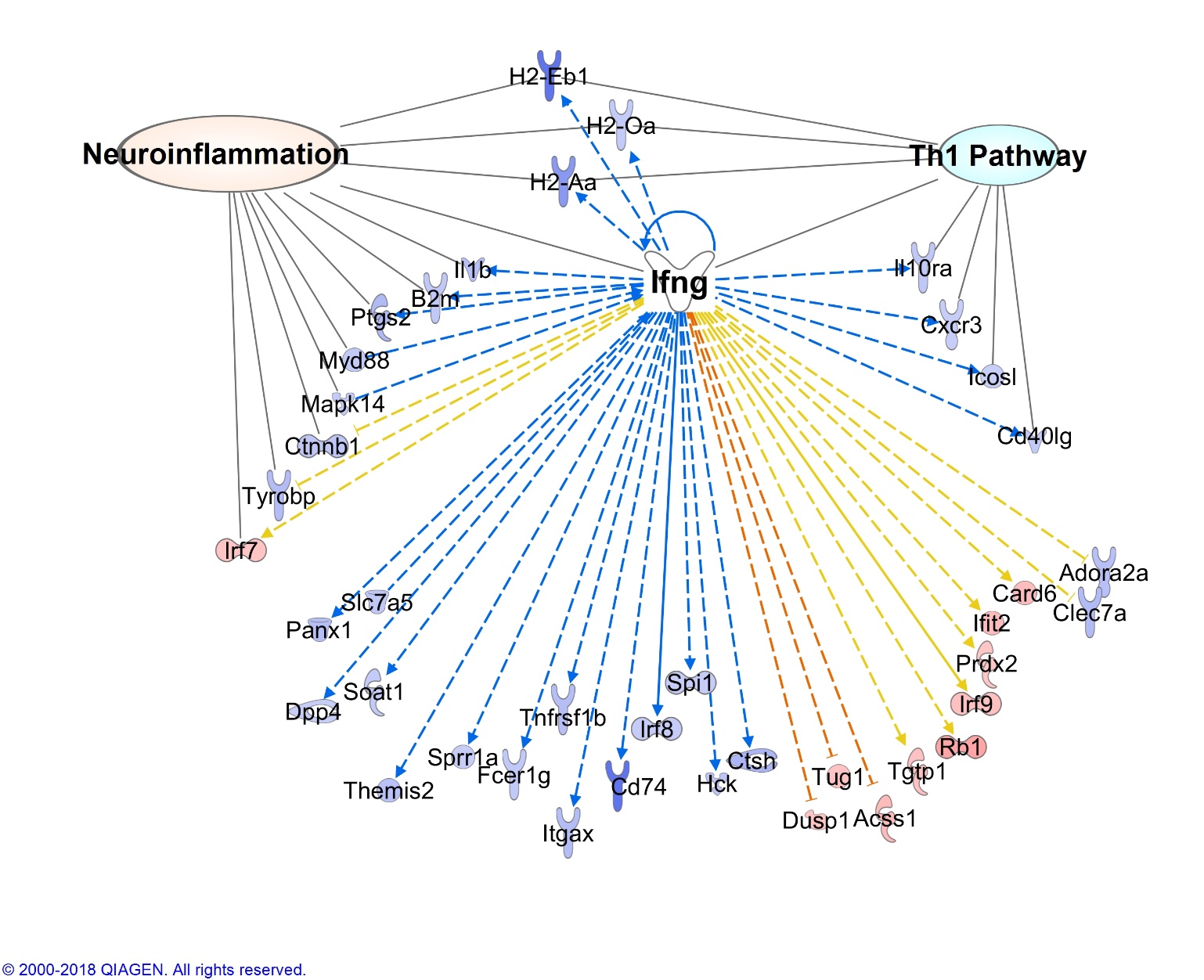
**

**Supplemental Figure 2. Bioinformatic analysis of key pathways regulated by VitD in Teff cells from B6 females.** A list of DE transcripts within the IFNγ node was imported into IPA Pathway Builder, and used to connect the following canonical pathways “Neuroinflammation Signaling Pathway” (Neuroinflammation) and “Th1 pathway” (Th1). Expression values are overlaid onto the molecules (Blue, downregulation by VitD; red, upregulation by VitD). Predicted regulation is indicated by arrow color (blue, upregulation; red/yellow downregulation.

**Supplemental Figure 3, part 1**

**
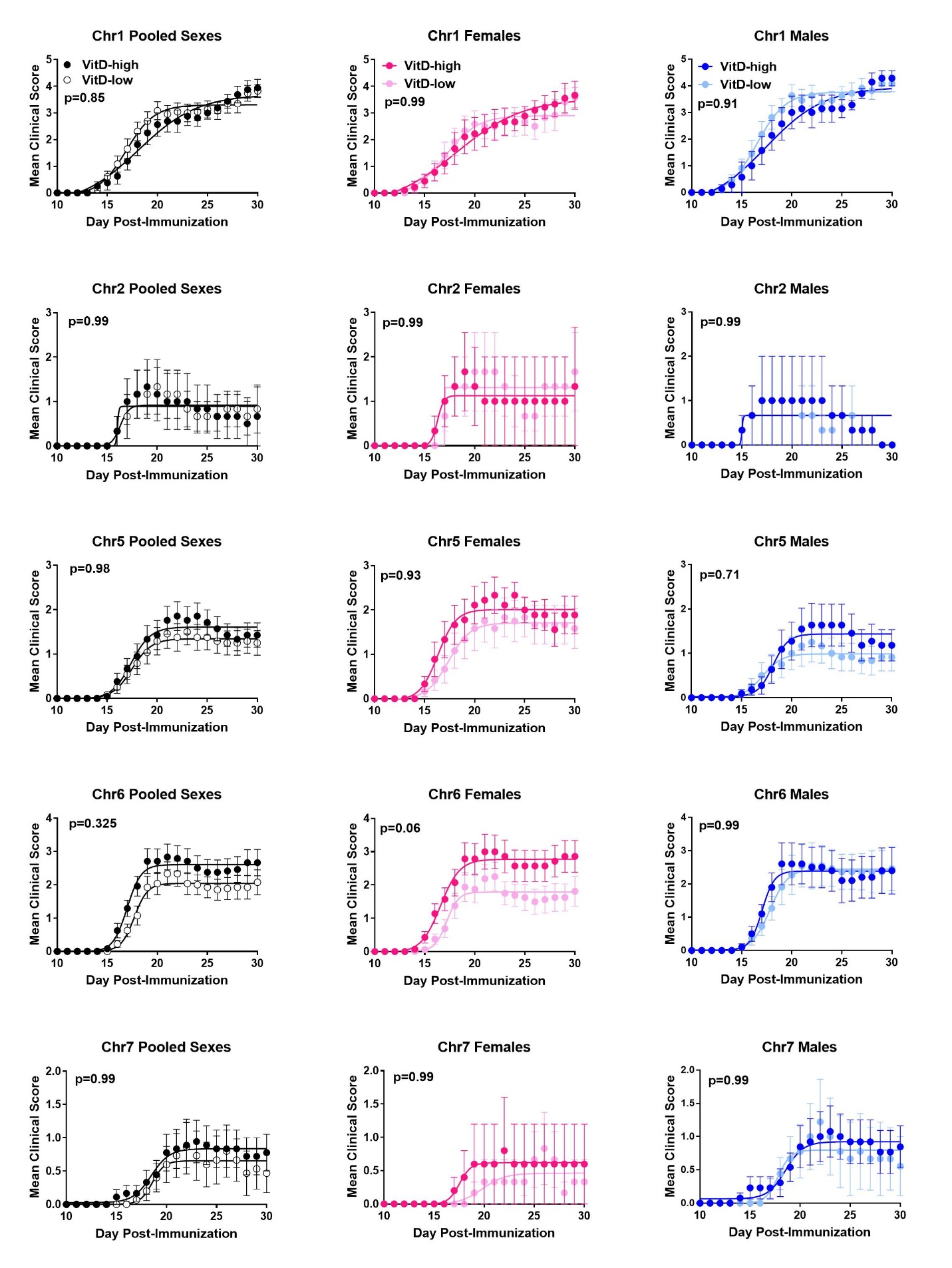
**

**Supplemental Figure 3, part 2**

**
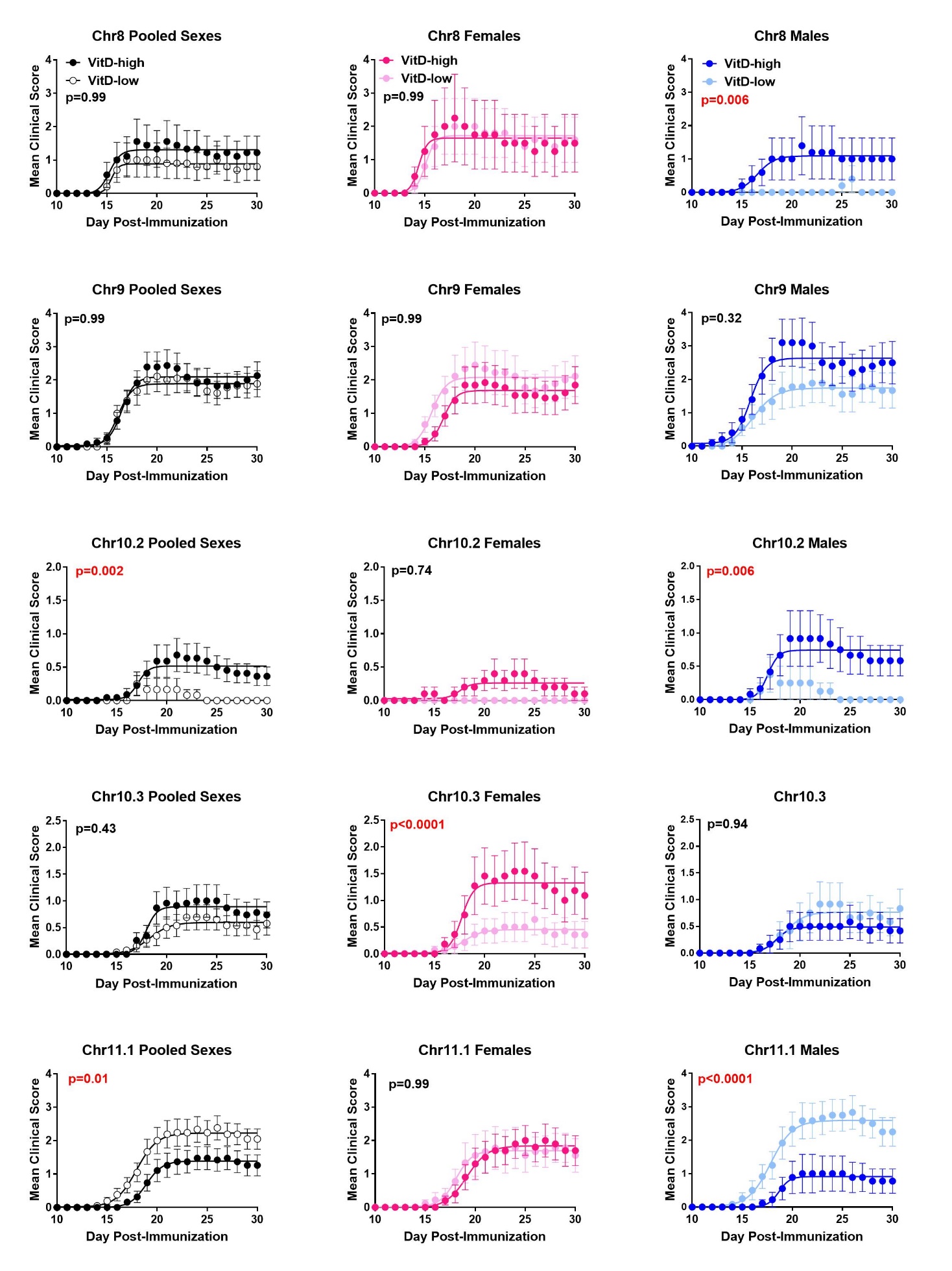
**

**Supplemental Figure 3, part 3
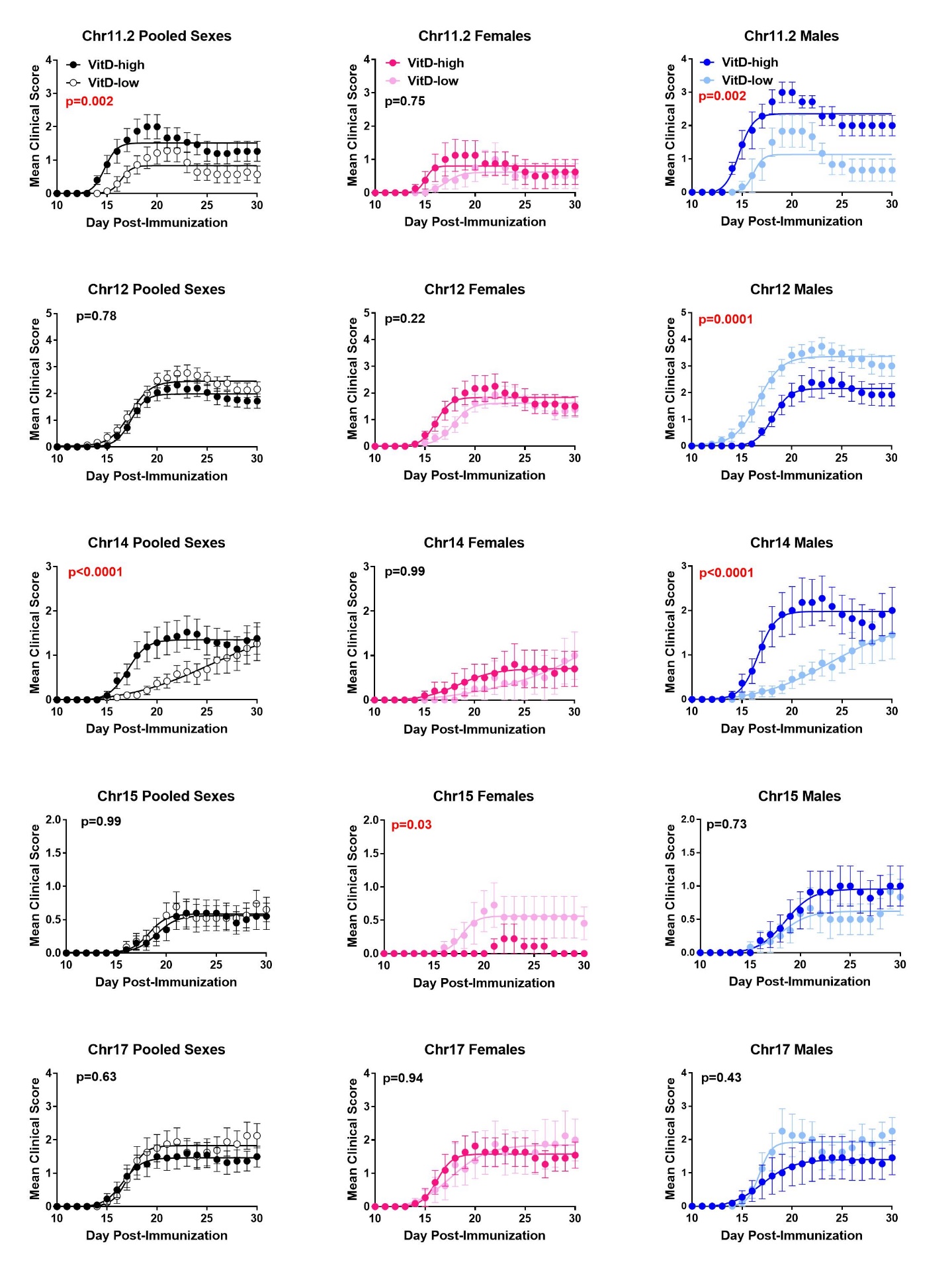
**

**Supplemental Figure 3, part 4
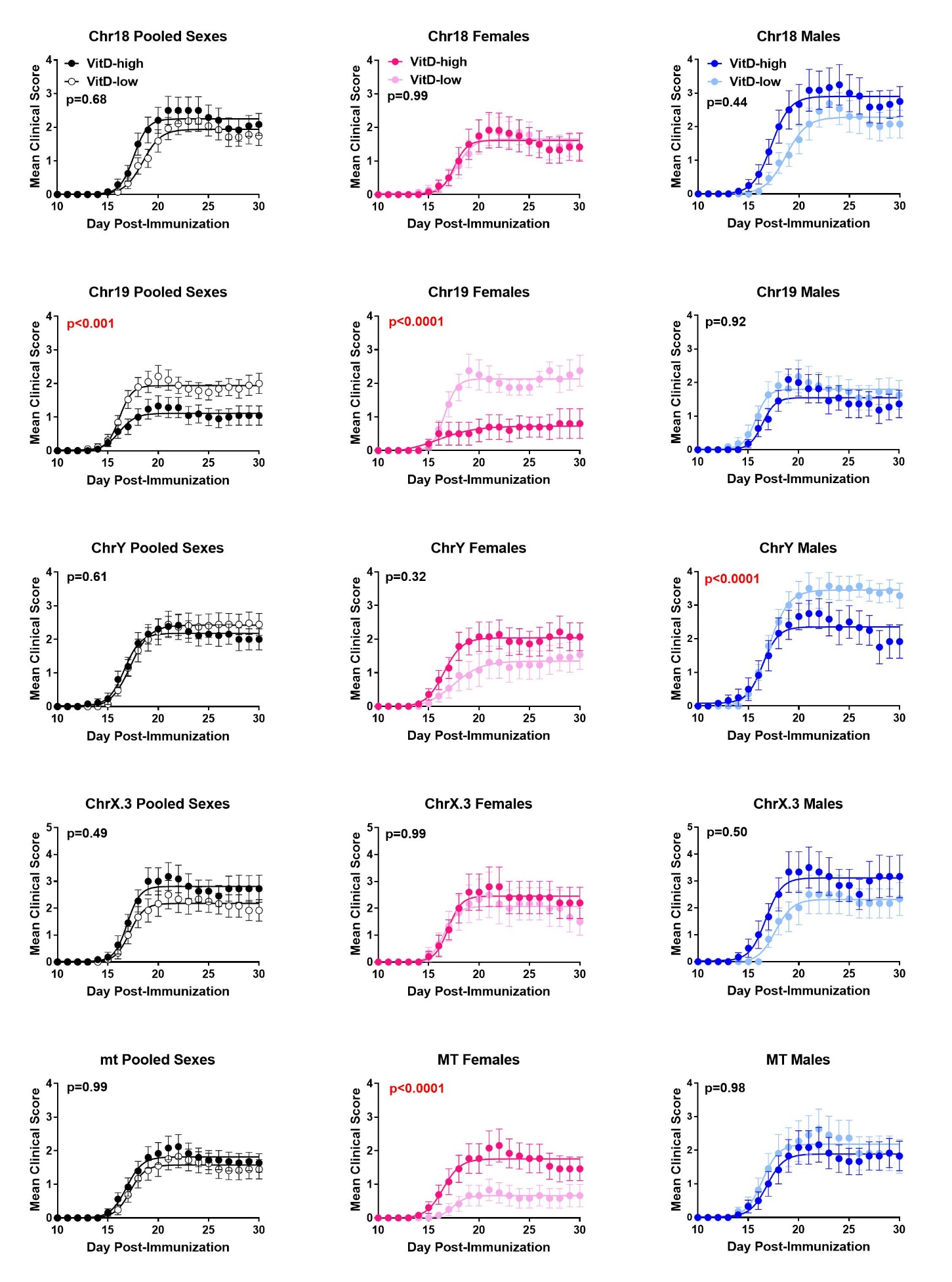
**

**Supplemental Figure 3. Effects of VitD on EAE disease course in** **B6.Chr^PWD^ consomic mice.** B6.Chr^PWD^ consomic mice were exposed to VitD-high and VitD-low diets, as in **Fig. 2**, followed by induction of EAE. Disease course for the indicated consomic strains is shown for pooled sexes, or for each sex separately, as designated. P-values for the effect of diet on overall EAE course (representing the interaction term for treatment × time, repeated measures 2-way ANOVA) are shown. Number of animals per group is provided in **Supplemental Table 1**.
